# FSTL1 promotes M2 polarization of macrophages via the DIP2A–Smad2/3 Axis in gastric cancer

**DOI:** 10.1186/s12967-026-08836-1

**Published:** 2026-08-21

**Authors:** Ming Yang, Rongfei Wang, Xiao Wang, Zheng Chen, Yonghui Su

**Affiliations:** Department of Gastrointestinal Surgery, The Fifth Affiliated Hospital, Sun Yat-Sen University, No. 52 Meihua East Road, Zhuhai, Guangdong 519000 P. R. China

**Keywords:** FSTL1, Gastric cancer, M2 polarization, DIP2A, TGF-β

## Abstract

**Background:**

Follistatin-like protein 1 (FSTL1) is aberrantly overexpressed in gastric cancer (GC) and modulates immune cell infiltration in the tumor microenvironment. However, the molecular mechanism underlying its regulation of macrophage polarization remains incompletely defined.

**Methods:**

In this study, we analyzed the expression of FSTL1 and its correlations with clinical prognosis and M2 macrophage infiltration using data from The Cancer Genome Atlas (TCGA) database and clinical GC tissue microarrays. In vitro experiments were conducted to validate the effect of FSTL1 on M2 polarization, and a mouse subcutaneous syngraft tumor model was established to substantiate its in vivo role. Combined analyses involving transcriptome sequencing, single-cell sequencing, and coimmunoprecipitation were performed to identify the key receptors and signaling pathways involved in FSTL1-mediated regulation.

**Results:**

The expression of FSTL1 was significantly upregulated in GC tissues, and its high expression was positively correlated with advanced clinical stage, unfavorable clinical outcomes, and increased M2 macrophage infiltration. In vitro, GC cell-derived FSTL1 markedly increased M2 polarization in both THP-1-derived M0 macrophages and monocyte-derived macrophages. This effect was significantly attenuated by *FSTL1* knockdown in GC cells and was restored in a dose-dependent manner by supplementation with exogenous recombinant FSTL1. In vivo, *Fstl1* knockdown suppressed syngeneic tumor growth, reduced the proportions of M2 macrophages and regulatory T cells, and increased the proportion of Cd8a⁺ T cells in tumor tissues. Mechanistically, FSTL1 was found to bind to its functional receptor DIP2A to induce Smad2/3 phosphorylation, thereby promoting macrophage M2 polarization, and blockade of either DIP2A or the TGF-β signaling pathway attenuated this effect. Notably, we identified a reciprocal positive feedback loop between GC cells and macrophages: tumor-derived FSTL1 activated the TGF-β/Smad2/3 pathway in macrophages to promote M2 polarization, and polarized M2 macrophages in turn secreted large amounts of TGF-β to further upregulate FSTL1 expression in GC cells. This positive feedback loop sustained M2 macrophage enrichment in the GC microenvironment, which reinforced the immunosuppressive landscape and contributed to the progression of tumors to malignancy.

**Conclusions:**

These findings indicate that FSTL1 represents a promising immunomodulatory target and highlight its potential as a therapeutic target to reprogram the immunosuppressive microenvironment and improve the efficacy of GC immunotherapy.

**Supplementary Information:**

The online version contains supplementary material available at https://doi.org/10.1186/s12967-026-08836-1.

## Introduction

Gastric cancer (GC) is the fifth most common malignant tumor worldwide, with both its incidence and mortality ranking at the forefront of all cancers [1]. Surgical resection remains the primary curative treatment for GC. However, most patients are diagnosed at an advanced stage and miss the optimal window for radical surgery, which creates an urgent unmet clinical need for novel therapeutic strategies [2, 3].

In recent years, immunotherapies involving immune checkpoint inhibitors (ICIs) have achieved substantial progress in GC treatment [4]. Nonetheless, the tumor microenvironment (TME) of GC is heavily infiltrated by immunosuppressive cells, including tumor-associated macrophages (TAMs), myeloid-derived suppressor cells (MDSCs), and regulatory T cells (Tregs), which collectively reduce the efficacy of immunotherapy [5–7]. Among these cell populations, TAMs are recognized as key cellular mediators of tumor immune evasion [8].

Monocytes/macrophages (M0 phenotype) can polarize into classically activated (M1) or alternatively activated (M2) macrophages in response to distinct microenvironmental cues, with opposing functional phenotypes [9]. M1 macrophages promote proinflammatory and antitumor immune responses, whereas M2 macrophages exert potent immunosuppressive effects through multiple mechanisms [10]. These M2 macrophages secrete anti-inflammatory cytokines such as interleukin-10 (IL-10) and transforming growth factor-beta (TGF-β) to directly suppress the cytotoxic activity of T cells and natural killer (NK) cells, thereby impairing antitumor immunity [11]. These cells also upregulate expression of immune checkpoint molecules such as PD-L1 on T cells to transduce inhibitory signals and promote T-cell exhaustion [12]. Additionally, they secrete chemokines such as C-C motif chemokine ligand 22 (CCL22) to recruit Tregs into the TME, further suppressing the effector functions of T cells [13]. Accordingly, reprogramming macrophage polarization within the TME has emerged as a promising strategy to increase the response rate to GC immunotherapy [14].

Follistatin-like protein 1 (FSTL1) is a secreted glycoprotein that was originally identified in mouse osteoblasts and was subsequently found to be expressed by multiple mesenchymal cell types, including fibroblasts and adipocytes [15, 16]. It is involved in a variety of signaling pathways and biological processes, including the regulation of inflammation, vascularization and immune homeostasis, and plays context-dependent biological roles in a variety of diseases, including its bidirectional regulatory roles in tumorigenesis [17]. It acts as a tumor promoter in hepatocellular carcinoma, GC, and prostate cancer but functions as a tumor suppressor in lung cancer, bladder cancer, and renal cell carcinoma—these divergent effects are likely determined by cell-type specificity and distinct signaling contexts within the TME [18]. In GC, FSTL1 expression is closely correlated with tumor malignancy and modulates the immunosuppressive status of the TME by regulating immune cell function, suggesting that it may be an important modulator in shaping the TME [19, 20]. However, whether FSTL1 is involved in regulating macrophage polarization remains incompletely characterized.

Our previous studies demonstrated a significant correlation between *FSTL1* expression and macrophage subset distribution in GC: *FSTL1* expression was inversely correlated with the proportion of M0 macrophages but positively correlated with the frequency of immunosuppressive M2 macrophages [21]. These findings suggest that FSTL1 may promote the polarization of TAMs toward the M2 phenotype, thereby contributing to the establishment of an immunosuppressive TME and ultimately attenuating antitumor immune responses and limiting the efficacy of immunotherapy. Building on these preliminary observations, the present study aimed to systematically elucidate the molecular mechanism through which FSTL1 regulates macrophage polarization, characterize a previously unrecognized axis of GC immune evasion, and identify a candidate immunomodulatory target to improve the response rate to immunotherapy in patients with GC.

## Materials and methods

### Bioinformatics analysis

The TIMER web server (https://compbio.cn/timer3/) was used to analyze *FSTL1* expression in GC and other tumor types, as well as its correlation with immune cell infiltration [22]. Transcriptomic profiles and matched clinical data for patients with GC were retrieved from The Cancer Genome Atlas (TCGA) database to assess differential *FSTL1* expression and its association with clinical stage, and to conduct Kaplan‒Meier survival analysis. All bioinformatic analyses were performed in R software (version 4.4.3). The single-cell RNA sequencing dataset GSE183904 was utilized to characterize *DIP2A* expression in macrophages and its correlation with M2 polarization scores in GC tissues. Myeloid cell clusters were extracted using myeloid cell marker genes (*LYZ* [lysozyme] and *CD68*), and macrophage clusters were further annotated using macrophage-specific marker genes (*CD68*, *CD163*, *MRC1* [mannose receptor C-type 1], and *C1QA* [complement C1q A chain]). *DIP2A* expression in macrophages was quantified, and M2 polarization scores for each macrophage were calculated based on the basis of the expression panel of M2 marker genes, including *CD163*, *MRC1*, *IL-10*, *TGFB1* [TGF-β1], *CCL18* [C-C motif chemokine ligand 18], *ARG1* [arginase 1], *C1QA*, and *C1QB* [complement C1q B chain]. Finally, uniform manifold approximation and projection (UMAP) plots for dimensionality reduction were generated to visualize the association between *DIP2A* expression and M2 polarization status in GC-infiltrating macrophages.

### Immunohistochemical (IHC) staining

IHC staining was performed on two independent tissue microarray cohorts purchased from Shanghai Outdo Biotech Co., Ltd.: cohort 1 included 29 paired GC and matched adjacent nontumor tissues, and cohort 2 included 74 paired samples, yielding a total of 103 paired tissues for analysis. Both cohorts were processed for staining and scoring under identical experimental conditions but in separate independent staining runs. Briefly, the paraffin sections were deparaffinized, rehydrated, and subjected to heat-induced antigen retrieval in sodium citrate buffer (pH 6.0). Endogenous peroxidase activity was quenched with 3% H_2_O_2_, followed by blocking with 10% normal goat serum to minimize nonspecific binding. The sections were then incubated overnight at 4 °C with primary antibodies against FSTL1 (20182–1-AP; Proteintech, China) or CD206 (18704–1-AP; Proteintech), followed by incubation with HRP-conjugated secondary antibodies for 1 h at room temperature. Peroxidase activity was visualized using 3,3’-diaminobenzidine (DAB) as the chromogen, and the sections were counterstained with hematoxylin and mounted with aqueous medium. FSTL1 expression was scored on the basis of both the proportion of positively stained tumor cells and the staining intensity [19]. Staining intensity was graded on a 4-point scale: 0 (negative), 1 (weak), 2 (moderate), and 3 (strong). The percentage of positive cells was scored as follows: 0 (0–5%), 1 (5–25%), 2 (25–50%), 3 (50–75%), and 4 (75–100%). The final immunoreactivity score was calculated as the product of the intensity score and the proportion score. Two blinded pathologists who were blinded to patient clinical information independently evaluated and recorded the staining results under a light microscope.

To eliminate technical batch variation from the two cohorts, the raw IHC scores of the tumor tissues were normalized via batchwise Z score transformation: Z=(X-X_batch_)/SD_batch_, where X denotes the raw IHC score and X_batch_ and SD_batch_ represent the mean and standard deviation, respectively, of the tumor IHC staining scores within each cohort. Unpaired t tests revealed significant interbatch differences in the raw FSTL1 IHC scores (*p* = 0.006). After batchwise Z score normalization, no statistically significant difference was observed between cohort 1 and cohort 2 for the FSTL1 Z scores (*p* > 0.999) and CD206 Z scores (*p* > 0.999), confirming the effective elimination of batch effects (Supplementary Figure 1A, B). Normalized Z scores were adopted for all subsequent statistical analyses.

### Cell culture and coculture

All cell lines were obtained from Fenghui Biotechnology Co., Ltd., China. The following cell lines were used in this study: the human normal gastric epithelial cell line GES-1, human GC cell lines (HGC27, MGC803, MKN45, and AGS), the mouse forestomach carcinoma cell line MFC, and the human monocytic cell line THP-1, which is inducible for macrophage differentiation.

GES-1, HGC27, MGC803, MKN45, MFC and THP-1 cells were maintained in complete RPMI-1640 medium (Gibco, USA) supplemented with 10% fetal bovine serum (FBS; Gibco) and 1% penicillin‒streptomycin (Beyotime, China). AGS cells were cultured in complete Ham’s F-12K medium (Servicebio, China) supplemented with 10% FBS and 1% penicillin‒streptomycin. All the cells were incubated at 37 °C in a humidified incubator (Thermo Fisher Scientific, USA) with 5% CO_2_.

Peripheral blood was collected from healthy donors in heparin-coated tubes after written informed consent was obtained. Peripheral blood mononuclear cells (PBMCs) were isolated via density gradient centrifugation using the commercial Human PBMC Separation Solution (Elabscience, China) according to the manufacturer’s protocol. Isolated PBMCs were resuspended in complete RPMI-1640 medium, seeded into 6-well plates at a density of 2 × 10^6^ cells/mL and incubated for 2 h at 37 °C with 5% CO_2_. Nonadherent cells were removed by gentle washing three times with prewarmed phosphate-buffered saline (PBS). Adherent monocytes were then cultured in complete RPMI-1640 medium supplemented with 50 ng/mL recombinant human macrophage colony-stimulating factor (M-CSF; Yeasen, China) for 7 days to allow them to differentiate into monocyte-derived macrophages (MDMs), with fresh medium replenished every 2–3 days. Prior to all coculture or drug treatment experiments, flow cytometric analysis targeting CD68 (333805; BioLegend, USA) was performed to characterize the purity and successful differentiation of mature MDMs (approximately 90%; Supplementary Figure 1J, K).

Transwell coculture assays were conducted using 24-well plates fitted with 0.4 μm pore size polycarbonate membrane inserts (Corning, USA). Briefly, THP-1 cells were seeded at a density of 1 × 10^5^ cells per well in the lower chamber with 600 μL complete medium containing phorbol 12-myristate 13-acetate (PMA, 50 ng/ml) to induce differentiation into M0 macrophages (designated THP-1_M0_ cells). The medium was replaced with fresh complete medium prior to coculture. MDMs prepared as described above were also seeded in the lower chamber at 1 × 10^5^ cells per well in 600 μL of complete medium. GC cells (5 × 10^4^ to 1 × 10^5^ cells per insert) were resuspended in 200 μL of low-serum medium and added to the upper chamber. All cocultures were incubated for 48 h at 37 °C with 5% CO_2_.

### Cell transfection and knockdown

Small interfering RNAs (siRNAs) targeting *FSTL1* and *DIP2A* were designed and chemically synthesized. MKN45 and THP-1 cells were transfected with the corresponding siRNAs using a lipid-mediated transfection method according to the experimental design. For stable knockdown, short hairpin RNAs (shRNAs) targeting *Fstl1* were cloned and inserted into a lentiviral expression vector. MFC cells were infected with the recombinant lentivirus, and stable knockdown cell lines were established via puromycin selection. All siRNA sequences and shRNA target sequences are listed in the Supplementary Tables.

### Flow cytometry assay

THP-1-derived macrophages or MDMs were collected, washed with PBS, and incubated with Fc receptor blocking reagent (Absin, China) for 30 min at 4 °C. Surface staining was performed with a CD11b-PerCp-Cy5.5 antibody (550993; BD Biosciences, USA) in prechilled PBS supplemented with 1% FBS for 30 min at 4 °C. After being washed, the cells were fixed with 200 μL of fixation buffer (420801; BioLegend) at room temperature for 30 min and then permeabilized with Intracellular Staining Permeabilization Wash Buffer (BioLegend). Intracellular staining was performed with a CD206-APC antibody (550889; BD Biosciences) for 30 min at room temperature in the dark. Finally, the cells were washed, resuspended in PBS, and analyzed on a flow cytometer.

### RNA extraction and quantitative real-time PCR (qRT‒PCR)

Total RNA was extracted using an RNA extraction kit (19231ES50; Yeasen). Complementary DNA (cDNA) was reverse transcribed from total RNA using a reverse transcription kit (11155ES60; Yeasen), and qRT‒PCR was performed with a qRT‒PCR kit (11201ES08; Yeasen) on an Applied Biosystems 7500 instrument. Human *GAPDH* or mouse *Gapdh* were used as endogenous reference genes. Relative mRNA expression levels of *FSTL1* (human), *Fstl1* (mouse) and *DIP2A* were calculated using the 2^(-ΔΔCt) method. All the primers used are listed in the Supplementary Tables.

### Western blotting (WB)

Total cellular protein was extracted using RIPA lysis buffer, and the protein concentration was determined via the Bradford assay. Protein samples were separated by sodium dodecyl sulfate‒polyacrylamide gel electrophoresis (SDS‒PAGE) and transferred onto polyvinylidene fluoride membranes (Millipore, USA). The membranes were blocked with 5% skim milk for 1 h at room temperature and then incubated overnight at 4 °C with primary antibodies, followed by incubation with horseradish peroxidase (HRP)-conjugated secondary antibodies for 1 h at room temperature. The protein bands were visualized using an iBright CL1500 imaging system (Invitrogen, USA). Primary antibodies against the following were used: FSTL1 (20182–1-AP), CD163 (68218–1-Ig), ARG1 (66129–1-Ig), NOS2 (18985–1-AP), and GAPDH (10494–1-AP) from Proteintech; Smad2/3 (8685) and phosphorylated Smad2/3 (p-Smad2/3; 8828) from Cell Signaling Technology (USA); and DIP2A (sc-293390) from Santa Cruz Biotechnology (USA).

### Reagent treatments and groupings

SB-431542 is a selective inhibitor of the TGF-β type I receptor (activin receptor-like kinase 5, ALK5). It acts by competitively binding to the ATP-binding pocket of the ALK5 intracellular kinase domain, thereby blocking downstream Smad2/3 phosphorylation and canonical TGF-β signal transduction. SB‑431542 (MedChemExpress, USA) was dissolved in dimethyl sulfoxide (DMSO) to prepare a 10 mM stock solution that was stored at −20 °C. Cell Counting Kit-8 (CCK-8) assays were performed to exclude the potential cytotoxicity of 1 μM SB-431542. Briefly, THP-1 cells were seeded at 5000 cells per well in 96-well plates and differentiated into M0 macrophages with PMA for 48 h. The cells were treated with 1 μM SB-431542 or an equivalent volume of DMSO (vehicle control), and cell viability was determined at 0 h and 48 h post-treatment. At each time point, 10 μL of CCK-8 reagent (Yesen) was added to each well, followed by incubation at 37 °C for 2 h. The optical density was measured at 450 nm, and the relative cell viability was calculated.

For in vitro functional assays, cells were pretreated with 1 μM SB‑431542 for 1 h prior to stimulation with recombinant human FSTL1 (rhFSTL1; HY-P70412; MedChemExpress) or recombinant human TGF-β1 (ab50036; Abcam, UK). An equivalent volume of DMSO was added to the control group to prevent solvent interference. After 48 h of culture, the cells were collected for subsequent flow cytometry and WB analyses.

### Coimmunoprecipitation (co-IP)

Co-IP assays were performed using the rProtein A/G IP/Co-IP Kit (Yeasen) in accordance with the manufacturer’s instructions. Briefly, THP-1 cells were collected and lysed with IP lysis buffer, after which the cell lysates were centrifuged at 14,000×g for 10 min at 4 °C, and the supernatant was collected for subsequent experiments. Pretreated magnetic beads were incubated with 2 μg of primary antibodies against FSTL1 or DIP2A (or isotype-matched IgG as a negative control) for 1 h at room temperature. After the antibody-bound beads were washed, the prepared cell lysate supernatant was added and incubated at 4 °C overnight with gentle rotation. Bound proteins were eluted by the addition of 100 μL of SDS‒PAGE loading buffer, followed by boiling at 95 °C for 5 min. The eluted supernatants were collected by magnetic separation and subjected to WB analysis to detect protein‒protein interactions. The VeriBlot for IP Detection Reagent (ab131366; Abcam) was used for immunodetection in these assays.

### Transcriptome sequencing and analysis

Total RNA was extracted from untreated THP-1_M0_ cells and THP-1_M0_ cells treated with 20 ng/mL rhFSTL1 for 48 h, after which DNBSEQ-T7 next-generation sequencing was performed. After quality control and normalization, read counts for each gene were obtained. The DESeq2 package (version 1.46.0) was used to identify differentially expressed genes (DEGs) between the two groups. Functional gene sets (including Gene Ontology [GO] and Kyoto Encyclopedia of Genes and Genomes [KEGG] pathways) were retrieved from the MSigDB database via the msigdbr package (version 25.1.1). GO enrichment analysis, KEGG pathway analysis, and gene set enrichment analysis (GSEA) were performed on the DEGs using the clusterProfiler package (version 4.14.6). Enrichment results were visualized using the enrichplot (version 1.26.6) and ggplot2 (version 4.0.1) packages.

### Animal experiments

Four- to six-week-old female C57BL/6 mice were randomly allocated to control and *Fstl1*-knockdown groups (*n* = 5 per group). Mice were subcutaneously injected with 2 × 10^6^ control MFC cells or stable *Fstl1*-knockdown MFC cells in the right flank. Tumor volume and mouse body weight were monitored every 5 days, and tumor volume was calculated using the formula V = 1/2 × length × width^2^, where length and width represent the longest and shortest diameters of the tumor, respectively. Twenty-five days after inoculation, the mice were euthanized by cervical dislocation, and the tumors were carefully excised and weighed. Paraffin-embedded tissue sections were subsequently prepared, and the proportion of CD206^+^ cells was assessed by IHC staining.

### Multiplex immunofluorescence (mIF) staining

Paraffin-embedded mouse tumor sections were deparaffinized and processed for heat-induced antigen retrieval in citrate buffer (pH 6.0). Endogenous peroxidase activity was quenched with a methanol-hydrogen peroxide solution. Sequential tyramide signal amplification (TSA)-based multiplex staining was performed for Cd8a (GB155692; Servicebio), Foxp3 (GB150152; Servicebio), and Tgfb1 (GB11119; Servicebio). Briefly, after incubation with HRP-conjugated secondary antibodies in each round, distinct fluorophore-conjugated tyramide reagents (iF488, iF555, and iF647) were applied for signal development. Antibody stripping buffer was used to remove bound primary and secondary antibodies after each TSA cycle. The sections were counterstained with DAPI, treated with an autofluorescence quenching reagent, and mounted with anti-fade mounting medium. Images were acquired using a digital tissue scanner. For quantification, three nonoverlapping random fields (400 × magnification) were captured per tumor section, avoiding necrotic or hemorrhagic areas. Cd8a^+^ and Foxp3^+^ cells were counted manually by an observer who was blinded to group allocation. The percentage of marker‑positive cells was calculated as the number of positively stained nuclei divided by the total number of DAPI^+^ nuclei. The mean of three fields was used as one biological replicate. Statistical comparisons between the shNC and sh*Fstl1*-3 groups (*n* = 5) were performed using an unpaired Student’s t test.

### ELISA

The concentration of TGF-β1 in the THP-1 cell culture supernatants was measured using a QuantiCyto® Human TGF-β1 ELISA Kit (EHC107b; Neobioscience, China) following the manufacturer’s protocol. Briefly, the samples were preactivated by mixing 11 μL of the supernatant with 79 μL of standard and sample diluent, followed by the addition of 10 μL of activation solution for 60 min at 2–8 °C, and termination with 10 μL of stop solution (10-fold dilution). A standard curve was generated via serial dilution of a reconstituted TGF-β1 protein standard, and 100 μL of activated samples or standards were added to microplate wells precoated with anti-TGF-β1 antibody and incubated for 90 min at 37 °C in the dark. After five washes, 100 μL of biotinylated anti-TGF-β1 antibody working solution was added and incubated for 60 min at 37 °C in the dark, followed by incubation with 100 μL of HRP-conjugated avidin working solution for 30 min at 37 °C in the dark after another wash. The chromogenic reaction was developed with 100 μL of TMB substrate for 15 min at 37 °C in the dark and then terminated with stop solution, and the optical density was measured at 450 nm within 3 min. The TGF-β1 concentration was calculated from the standard curve and corrected for the 10-fold dilution factor, with all assays performed in duplicate.

### Statistical analysis

The normality of the data distribution was assessed using the Shapiro–Wilk test prior to all the parametric statistical analyses. Data are shown as the mean ± standard deviation (SD) and interquartile range (IQR), as indicated in each figure legend. For comparisons between two independent groups with normally distributed data, the unpaired two-tailed Student’s t test was applied. For experiments involving multiple treatment groups, each treatment group was compared individually to its corresponding control group using an unpaired Student’s t test. All experiments were performed with at least three independent biological replicates, unless otherwise specified. All the statistical analyses were conducted using GraphPad Prism 8.0 (San Diego, CA). All exact *p* values are provided in Supplementary Table 4. *p* < 0.05 was considered to indicate statistical significance: *p < 0.05, **p < 0.01, ***p < 0.001, and #*p* > 0.05.

## Results

### *FSTL1* is aberrantly overexpressed in GC and is correlated with poor outcomes

**Fig. 1:**
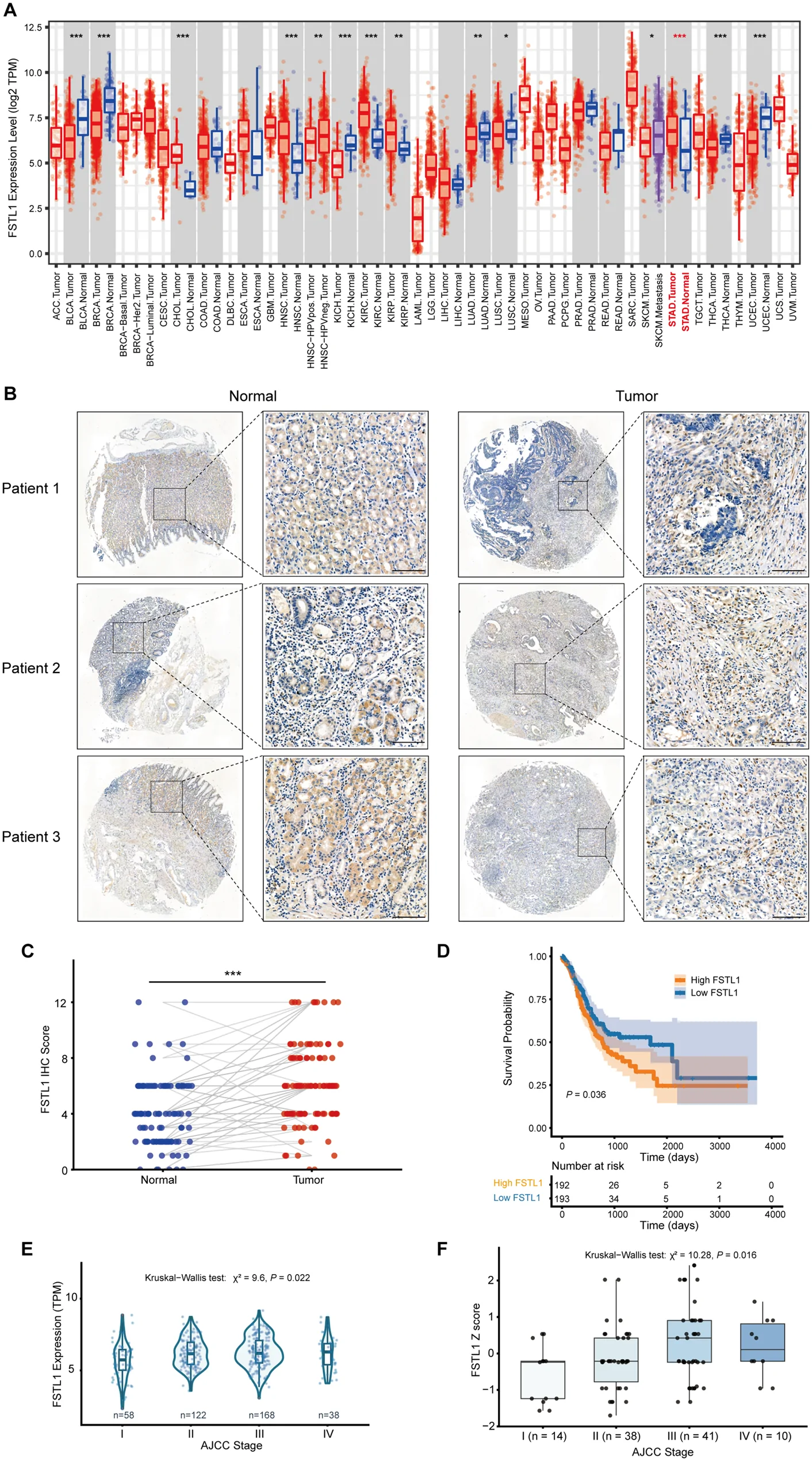
*FSTL1* is aberrantly overexpressed in GC and is correlated with poor clinical outcomes. (**A**) *FSTL1* expression levels across diverse cancer types were analyzed via the TIMER web server, with red denoting tumor tissues and blue denoting normal tissues. (**B**) Representative IHC staining images of FSTL1 in 3 paired GC tissues and matched adjacent non‑tumor tissues; scale bar = 100 μm. (**C**) IHC staining scores of FSTL1 in 103 paired GC tissues and matched adjacent non‑tumor tissues. The grey lines indicate individual matched sample pairs. Statistical significance was determined by the paired Wilcoxon signed-rank test. (**D**) Kaplan‒Meier survival curves of GC patients in the TCGA-STAD cohort with high versus low levels of *FSTL1* expression. Differences in survival between the two groups were evaluated by the log-rank test. (**E**) Comparison of the *FSTL1* expression levels among patients with different AJCC stages in the TCGA-STAD cohort. (**F**) Correlations between AJCC stages and FSTL1 Z scores in 103 GC patients. Statistical significance was determined by the Kruskal‒Wallis test with Dunn’s post hoc test. The box plots in (**A**, **E**, **F**) show the median (central line) and IQR (box boundaries), and the whiskers represent 1.5‑fold of the IQR

The TIMER web server was used to evaluate *FSTL1* expression levels across diverse cancer types. *FSTL1* expression significantly downregulated in tumors such as bladder cancer and breast cancer, whereas it was markedly elevated in multiple tumor types, including GC (Fig. 1A). Kaplan‒Meier survival analysis of data from the TCGA-STAD cohort revealed that GC patients with high *FSTL1* expression had poorer clinical outcomes (*p* = 0.036; Fig. 1D).

IHC staining was performed to assess FSTL1 expression in 103 pairs of GC tissues and their matched adjacent normal tissues across two independent cohorts. FSTL1 expression was significantly higher in GC tissues than in paired adjacent nontumor tissues across the combined cohort (Fig. 1B, C). The TCGA data revealed that *FSTL1* expression was lower in patients with stage I GC than in those at more advanced clinical stages (Fig. 1E). Consistent with this trend, IHC staining analysis of the pooled cohort revealed that patients with early-stage GC had lower FSTL1 Z scores (Fig. 1F), suggesting that FSTL1 may contribute to the malignant progression of GC.

Further TCGA-STAD cohort analysis via cBioPortal revealed that somatic mutation of *FSTL1* only occurred in only 0.7% of GC patients with no functional driver variants, indicating that *FSTL1*-driven GC progression is primarily mediated through transcriptional upregulation rather than genomic mutation [23].

### *FSTL1* modulates M2 macrophage infiltration in the TME

**Fig. 2:**
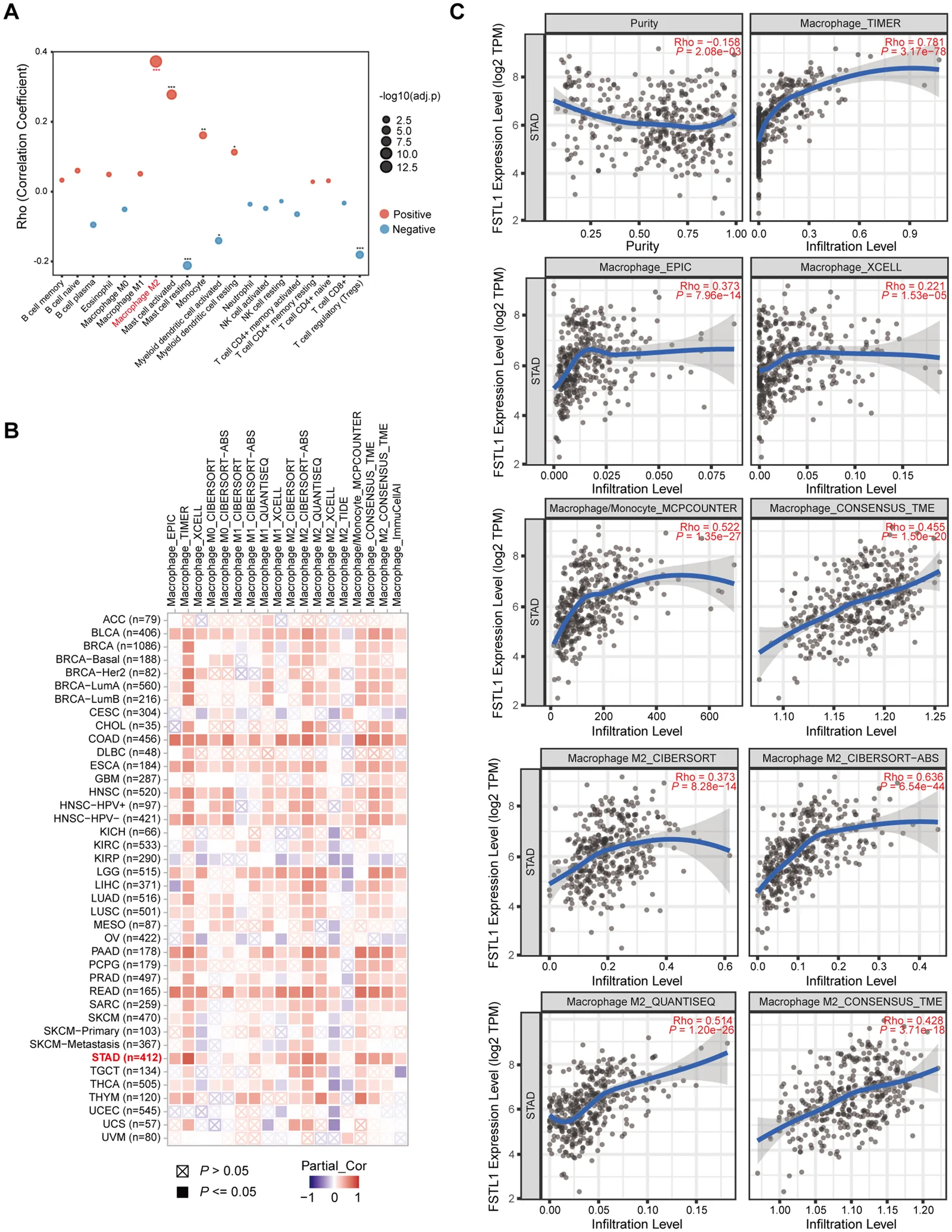
*FSTL1* is associated with immune cell infiltration profiles in the TME. (**A**) Correlations between the abundance of diverse immune cell subsets and *FSTL1* expression in the TME were analyzed using the TIMER web server. (**B**) Correlations between multiple macrophage signature scores and *FSTL1* expression across cancer types, with red indicating a positive correlation and blue indicating a negative correlation. (**C**) Scatter plots illustrating the correlations between the scores of multiple macrophage types and *FSTL1* expression in GC

Our previous studies revealed that *FSTL1* is associated with immune infiltration in GC, with a particularly strong correlation with macrophage infiltration [21]. To further verify this correlation, the relationship between *FSTL1* expression and the proportion of immune cells in the TME was analyzed using the TIMER web server. The abundances of M2 macrophages, activated mast cells, monocytes, and resting myeloid dendritic cells were positively correlated with *FSTL1* expression, whereas those of resting mast cells, resting myeloid dendritic cells and Tregs were negatively correlated. Among all the immune cell subsets assessed, M2 macrophages displayed the strongest and most significant positive correlation with *FSTL1* expression (Fig. 2A).

We further analyzed the correlations between *FSTL1* expression and multiple macrophage signature scores across different cancer types. *FSTL1* expression was positively correlated with most macrophage scores, even in different cancer types (Fig. 2B). Specifically, in GC, *FSTL1* expression was negatively correlated with tumor purity but positively correlated with most macrophage scores and M2 macrophage scores (Fig. 2C). Collectively, these results strongly indicate that *FSTL1* expression is associated with the M2 polarization of macrophages.

**Fig. 3:**
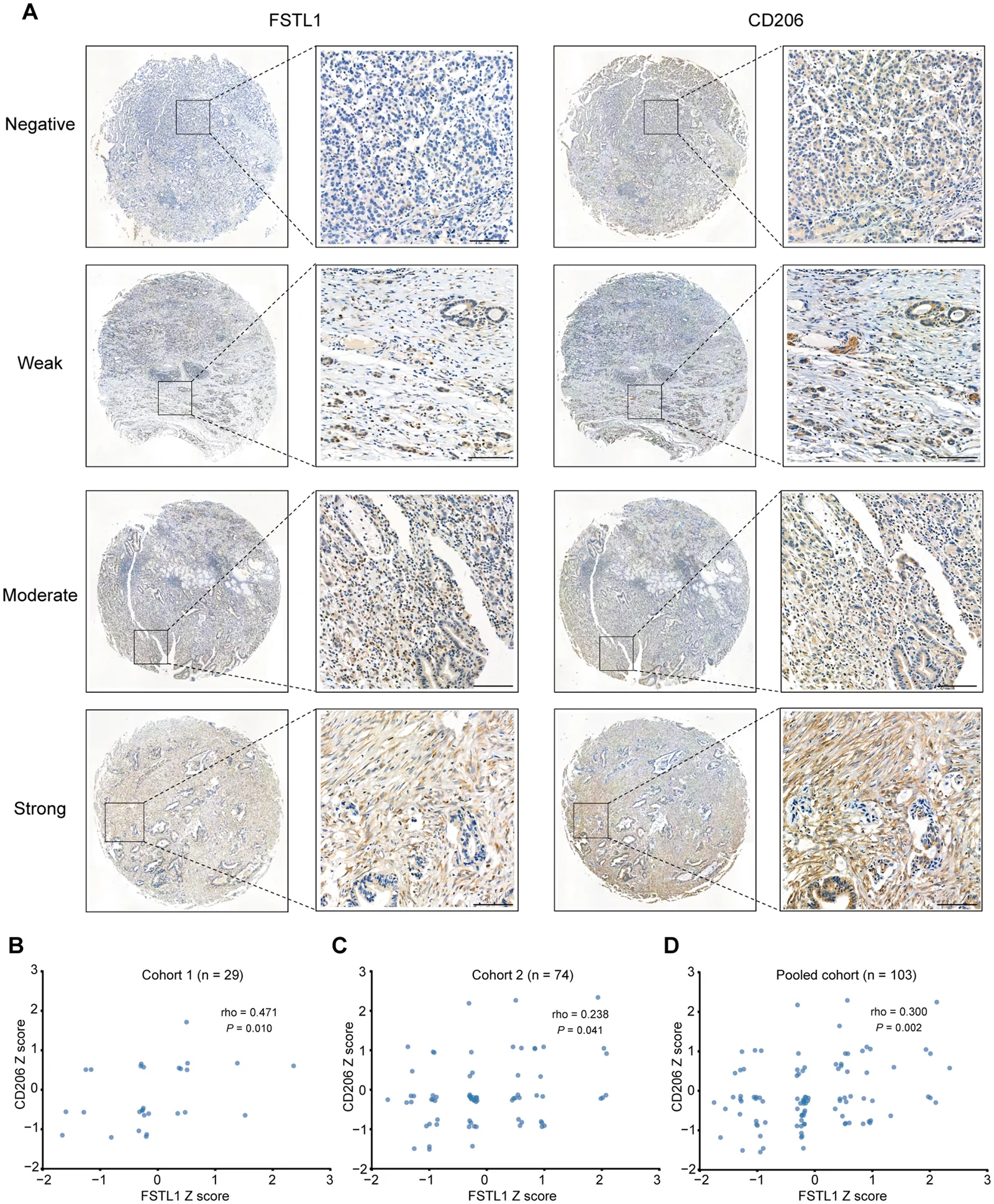
FSTL1 expression is positively associated with M2 macrophage infiltration in GC. (**A**) Representative IHC staining images of FSTL1 and CD206 in a 103-patient GC tissue cohort; scale bar = 100 μm. (**B, C**) Spearman correlation of FSTL1 and CD206 IHC Z scores in cohort 1 (*n* = 29) and cohort 2 (*n* = 74). (**D**) Spearman correlation of FSTL1 and CD206 IHC staining Z scores in the pooled cohort (*n* = 103). Spearman’s rank‑order correlation test was used to evaluate the correlation, and rho represents the Spearman correlation coefficient

To validate this at the protein level, we stained our GC cohort for CD206, a canonical M2 macrophage marker. Spearman correlation analyses were performed separately in two independent subcohorts, both of which revealed a positive association between FSTL1 and CD206 (Fig. 3B, C). Pooled analysis confirmed a significant positive correlation (rho = 0.300, *p* = 0.002; Fig. 3D), indicating that tumors with higher levels of FSTL1 expression harboured more CD206^+^ M2 macrophages.

### FSTL1 promotes M2 polarization of macrophages in vitro

**Fig. 4:**
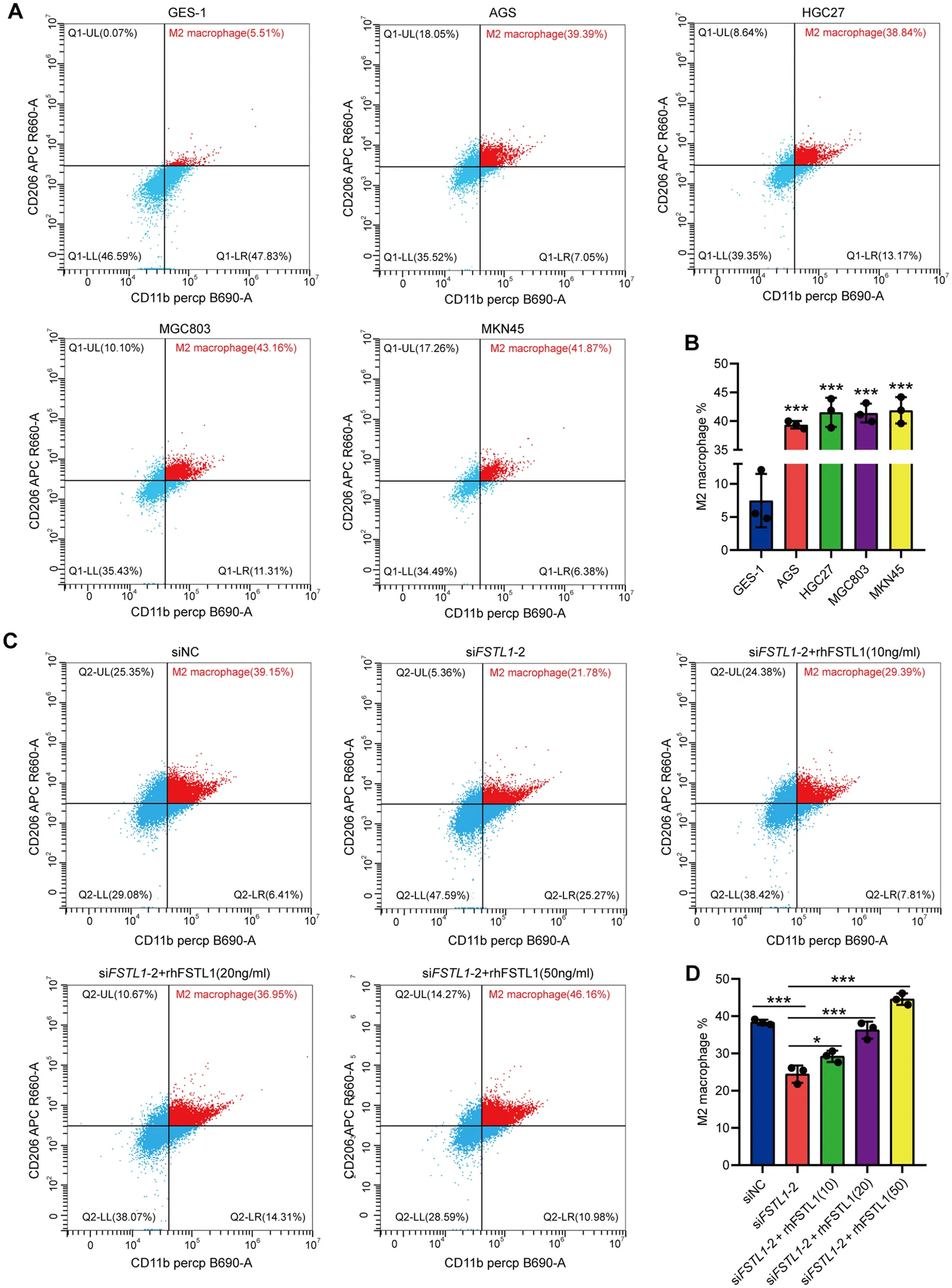
FSTL1 drives M2 polarization of macrophages in vitro. (**A**) Flow cytometric analysis of the proportion of CD206^+^ M2-like macrophages in THP‑1_M0_ cells cocultured with GES‑1, AGS, HGC27, MGC803, or MKN45 cells. (**B**) Quantification of the flow cytometric results shown in (**A**). (**C**) Flow cytometric analysis of CD206^+^ M2-like macrophage proportions in THP‑1_M0_ cells co‑cultured with siNC‑ or si*FSTL1*‑2‑transfected MKN45 cells or si*FSTL1*‑2‑transfected MKN45 cells supplemented with 10, 20, or 50 ng/mL rhFSTL1. (**D**) Quantification of the flow cytometric results shown in (**C**). The data in (**B, D**) Are presented as the mean ± SD from three independent biological experiments (*n* = 3). Each treatment group was compared individually to its respective control group using an unpaired Student’s t test

In our previous work, we confirmed that *FSTL1* expression was significantly elevated in GC cell lines compared with that in the normal gastric epithelial cell line GES-1, with MKN45 cells exhibiting the highest expression level [21]. Transwell coculture assays were performed to explore the effect of GC cells on macrophage polarization. Compared with coculture with GES-1 cells, coculture with all four GC cell lines markedly increased the polarization of THP-1_M0_ cells toward CD206^+^ M2 macrophages (Fig. 4A, B).

We next used siRNA to knockdown *FSTL1* expression in MKN45 cells. The results of qRT‒PCR and WB revealed that si*FSTL1*-2 achieved the highest knockdown efficiency (Supplementary Figure 1D, E). We then repeated the Transwell coculture assays by seeding *FSTL1*-knockdown or control MKN45 cells in the upper chamber. After 48 h of coculture, flow cytometry analysis revealed that the proportion of CD206^+^ cells was significantly reduced in the *FSTL1*-knockdown group. The addition of exogenous rhFSTL1 to the lower chamber restored this phenotype in a dose-dependent manner (Fig. 4C, D). These findings confirm that FSTL1 is a functional mediator that drives M2 polarization of macrophages.

To verify whether this regulatory effect is recapitulated in primary human macrophages, we isolated human PBMCs and differentiated them into MDMs for parallel coculture experiments. Consistent with the observations in THP-1-derived macrophages, the flow cytometry results revealed that coculture with GC cells significantly increased the percentage of CD206^+^ M2-like MDMs (Supplementary Figure 2A, B), whereas *FSTL1* depletion in GC cells largely attenuated this phenotypic shift, which could be reversed by exogenous rhFSTL1 supplementation (Supplementary Figure 2C, D). Taken together, these findings confirm that FSTL1 functions as a driver of M2 macrophage polarization.

### Fstl1 potentiates M2 polarization of macrophages in vivo

Next, we designed three lentiviral shRNAs targeting *Fstl1* and used them to infect MFC cells. The results of qRT‒PCR and WB revealed that all three shRNAs effectively knocked down *Fstl1* expression, among which sh*Fstl1*-3 had the highest knockdown efficiency (Supplementary Figure 1F, G). We established a stable *Fstl1*-knockdown MFC cell line based on sh*Fstl1*-3 and generated a subcutaneous syngeneic mouse model by inoculating control and *Fstl1*-knockdown MFC cells. Tumor growth was significantly slowed in the *Fstl1*-knockdown group. The mice were sacrificed 25 days after inoculation, and tumor tissues were collected for IHC and mIF staining. The densities of CD206^+^ cells in tumor tissues were significantly lower in the *Fstl1*-knockdown group than in the control group.

**Fig. 5:**
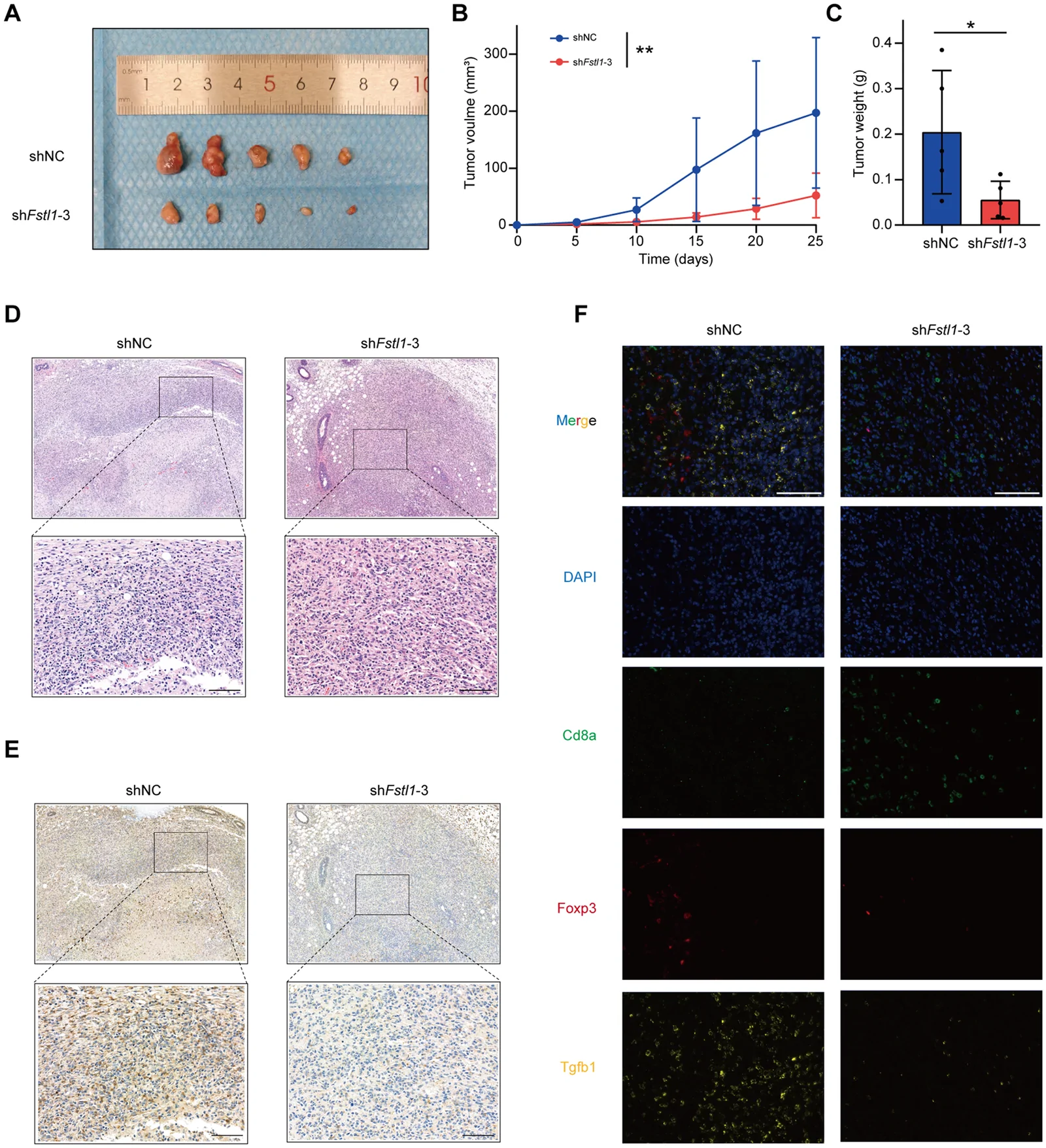
Fstl1 drives M2 polarization of macrophages in vivo. (**A**) Gross images of subcutaneous syngeneic tumors in C57BL/6 mice 25 days after the inoculation of control shNC or stable *Fstl1*‑knockdown MFC cells. (**B**) Tumor growth curves of the control and *Fstl1*‑knockdown groups. Statistical significance was determined by two-way repeated-measures ANOVA to evaluate overall differences in tumor growth kinetics between groups. (**C**) Final tumor weights in the two groups. Statistical significance was determined by the unpaired Student’s t test. (**D**) Representative H&E staining images of mouse tumor tissues. (**E**) Representative IHC staining images of CD206 in mouse tumor tissues. (**F**) Representative mIF staining images of Cd8a, Foxp3, and Tgfb1 in mouse tumor tissues. The data in (**B, C**) Are presented as the mean ± SD (*n* = 5 per group); all scale bars = 100 μm

To further characterize the intratumoral immune landscape, mIF staining (DAPI/Cd8a/Foxp3/Tgfb1) was performed on harvested tumor tissues. We observed that *Fstl1* depletion increased the infiltration of Cd8a^+^ cytotoxic T cells and reduced the accumulation of Foxp3^+^ regulatory T cells, further indicating that Fstl1 promotes an immunosuppressive TME in vivo (Fig. 5F & Supplementary Figure 4A).

### The pro-polarization function of FSTL1 is mediated by the TGF-β signaling pathway

**Fig. 6:**
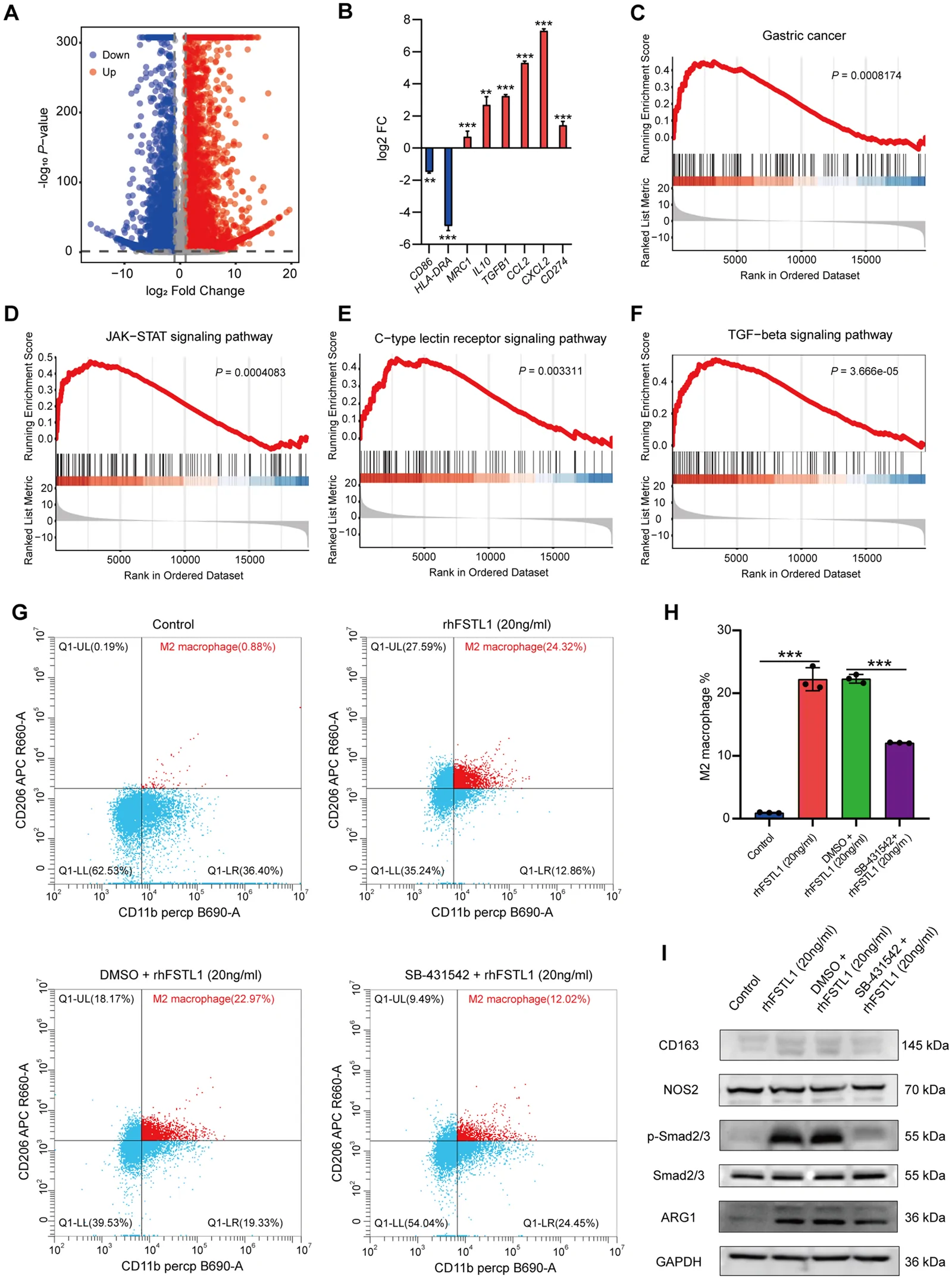
FSTL1 promotes M2 macrophage polarization via the TGF-β/Smad2/3 signaling pathway. (**A**) Volcano plot illustrating DEGs in the FSTL1‑treated THP‑1_M0_ cells relative to the control cells, with red indicating genes with upregulated expression and blue indicating genes with downregulated expression. (**B**) Relative expression levels of M1‑related and M2‑related genes and chemokines in the FSTL1-treated THP‑1_M0_ cells. GSEA plots showing significant positive enrichment of the GC pathway (**C**), JAK‑STAT signaling pathway (**D**), C‑type lectin receptor signaling pathway (**E**), and TGF‑β signaling pathway (**F**). (**G, H**) Flow cytometric analysis of the effect of SB‑431542 pretreatment on the FSTL1-induced M2 polarization of THP‑1_M0_ cells. The data in (**B, H**) Are presented as the mean ± SD from three independent biological experiments (*n* = 3), and statistical significance was determined by an unpaired Student’s t test. (**I**) WB analysis of the effects of SB‑431542 pretreatment on FSTL1‑induced Smad2/3 phosphorylation and M1/M2 marker expression in THP‑1_M0_ cells. For the transcriptomic sequencing data (**A**–**F**), differential expression and enrichment significance were calculated using standard bioinformatics algorithms

To explore the downstream mechanism underlying FSTL1-induced M2 polarization, we performed RNA sequencing on rhFSTL1-treated THP-1_M0_ cells and conducted differential gene expression analysis (Fig. 6A & Supplementary Figure 2A). Compared with the control group, the FSTL1-treated group exhibited downregulated expression of M1-related genes (such as *CD86* and *HLA-DRA)* but significantly upregulated expression of M2 marker genes and canonical M2-associated cytokines (*MRC1, IL10*, and *TGFB1*). The expression of several chemokines (such as *CCL17*, *CCL22* and *CD274* [programmed death-ligand 1, also known as PD-L1]) was also significantly increased, suggesting that FSTL1 may remodel the macrophage transcriptional profile to support an immunosuppressive TME (Fig. 6B).

Enrichment analysis further revealed that the DEGs were enriched mainly in immune- and inflammation-related signaling pathways (Supplementary Figure 3B). GO enrichment analysis revealed that the DEGs were significantly enriched in biological processes including cell chemotaxis, leukocyte migration and adhesion, and cytokine production, suggesting that FSTL1 may modulate the TME by regulating immune cell migration and function (Supplementary Figure 3C). GSEA further confirmed that M2-related signaling pathways were activated in THP-1_M0_ cells following FSTL1 treatment (Fig. 6D, E, and Supplementary Figure 3D–G). Notably, the GC pathway and TGF-β pathway were also significantly enriched (Fig. 6C, F). Given that FSTL1 is a known TGF-β-induced protein [24] and that the expression of *TGFB1* (the gene encoding TGF-β1) was significantly upregulated in FSTL1-treated macrophages (Fig. 6B), we hypothesized that FSTL1 promotes M2 polarization, at least in part, by activating the canonical TGF-β/Smad2/3 signaling pathway, with TGF-β1 acting as a key effector ligand.

Prior to functional intervention experiments, a CCK‑8 viability assay was performed to exclude the cytotoxic effect of SB‑431542. No obvious difference in relative cell viability was observed between the 1 μM SB‑431542‑treated group and the DMSO vehicle group at baseline (0 h) or after 48 h of incubation (Supplementary Figure 1C).

To validate this hypothesis, THP‑1_M0_ cells were pretreated with 1 μM SB‑431542 for 1 h prior to rhFSTL1 stimulation, with DMSO used as a vehicle control. Compared with the control treatment, the rhFSTL1 treatment significantly increased the proportion of CD206^+^ cells, which was accompanied by elevated protein levels of p-Smad2/3 and the canonical M2 markers CD163 and ARG1. The FSTL1-induced increase in CD206^+^ cells and p-Smad2/3, CD163, and ARG1 expression was partially abrogated by SB-431542 pretreatment. Notably, the expression of NOS2, a representative M1 macrophage marker, did not significantly change across all the experimental groups (Fig. 6G–I & Supplementary Figure 4D). Consistent with the findings in THP-1 cells, our results revealed that rhFSTL1 treatment significantly increased the percentage of CD206^+^ M2-like MDMs, and this phenotypic induction was markedly attenuated by SB-431542 pretreatment (Supplementary Figure 4B, C). These results indicate that FSTL1 drives macrophages toward the M2 phenotype in a TGF‑β/Smad2/3‑dependent manner.

### DIP2A serves as the functional receptor mediating FSTL1-induced M2 polarization

Previous studies have reported that the biological functions of FSTL1 are typically mediated through the cell-surface receptor DIP2A [20, 25, 26]. Given our above finding that FSTL1 acts via the TGF‑β/Smad2/3 pathway (with TGF‑β1 as the major effector), we next investigated whether DIP2A serves as the upstream functional receptor that links FSTL1 to TGF‑β1 secretion and Smad2/3 phosphorylation.

**Fig. 7:**
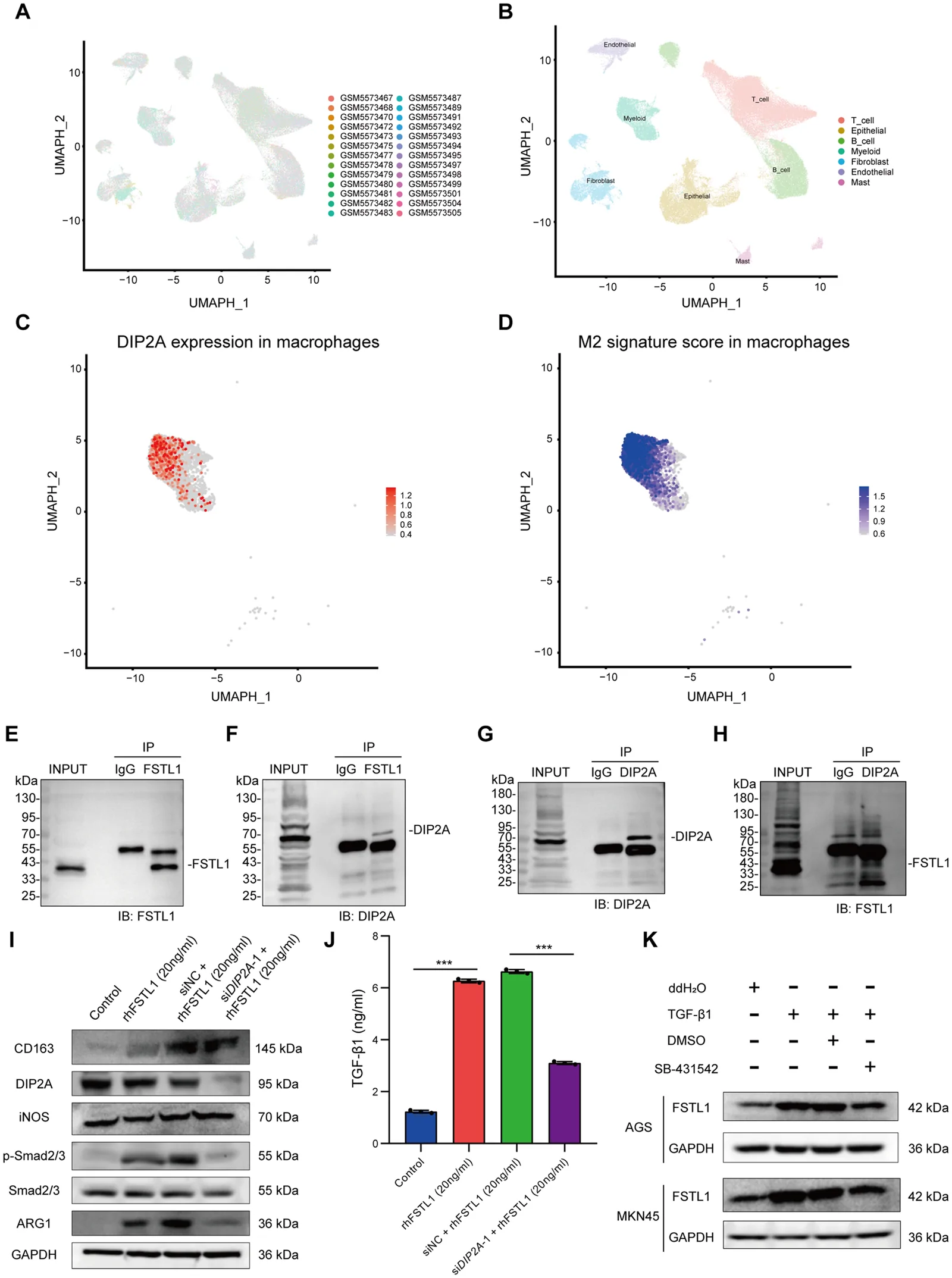
DIP2A serves as the functional receptor for FSTL1 in macrophages. (**A**) UMAP dimensionality reduction and clustering plot of all cells from GC samples in GSE183904, colored by sample. (**B**) UMAP plot with cell‑type annotations, including T cells, epithelial cells, B cells, myeloid cells, fibroblasts, endothelial cells and mast cells. (**C**) UMAP plot showing *DIP2A* expression in macrophage subsets, with darker red representing higher expression levels. (**D**) UMAP plot showing M2 signature scores in macrophage subsets, with darker blue representing higher scores. (**E**–**H**) Co‑IP assays validating the direct physical interaction between FSTL1 and DIP2A in THP‑1_M0_ cells. (**I**) WB analysis of the effect of si*DIP2A*‑1 on FSTL1‑induced Smad2/3 phosphorylation and M1/M2 marker expression. (**J**) ELISA-based quantification of TGF‑β1 concentrations in culture supernatants of FSTL1‑treated THP‑1_M0_ cells and the effect of si*DIP2A*‑1 on FSTL1‑induced TGF‑β1 secretion. Data are presented as mean ± SD from three independent biological experiments (*n* = 3). Statistical significance was determined using an unpaired Student’s t test. (**K**) AGS and MKN45 cells were treated with recombinant human TGF-β1 (10 ng/mL) for 48 h. WB analyses demonstrated that TGF-β1 treatment significantly upregulated FSTL1 expression at the protein level in both cell lines. This inductive effect was substantially attenuated by pretreatment with SB-431542

We first analyzed *DIP2A* expression in GC tissues using single-cell RNA sequencing data, which revealed that *DIP2A* was highly expressed in TAMs. Moreover, macrophages with higher *DIP2A* expression also exhibited higher M2 scores (Fig. 7A–D). Furthermore, Co-IP assays confirmed the direct physical interaction between FSTL1 and DIP2A in THP-1 cells (Fig. 7E–H). We then used siRNA to silence *DIP2A* expression in THP-1_M0_ cells. The results of the qRT‒PCR and WB analyses revealed that si*DIP2A*-1 exhibited the highest knockdown efficiency (Supplementary Figure 1 H, I). Silencing *DIP2A* in THP-1_M0_ cells significantly inhibited the FSTL1-induced increase in Smad2/3 phosphorylation (Fig. 7I). Consistent with these findings, the results of the WB analysis of macrophage polarization markers revealed that *DIP2A* silencing reversed the FSTL1-mediated upregulation expression of the M2 markers CD163 and ARG1, while the expression of the M1 marker NOS2 did not obviously change across all the experimental groups (Fig. 7I .& Supplementary Figure 4E).

### The TGF-β/FSTL1 reciprocal positive feedback loop

We performed ELISA to measure TGF-β1 the concentrations of in the culture supernatants of THP-1_M0_ cells. FSTL1 treatment significantly increased TGF-β1 secretion by THP-1_M0_ cells, whereas *DIP2A* knockdown with si*DIP2A*-1 effectively reversed the promoting effect of FSTL1 on TGF-β1 secretion (Fig. 7J). These results indicate that DIP2A is a functional receptor through which FSTL1 activates the TGF-β/Smad2/3 signaling pathway, promotes TGF-β1 secretion, and ultimately drives the M2 polarization of macrophages.

Given that polarized macrophages secrete high levels of TGF-β1, we next explored whether TGF-β1 could reciprocally modulate FSTL1 expression in GC cells to construct a closed-feedback circuit. We treated AGS and MKN45 GC cell lines with exogenous recombinant TGF-β1. WB and qRT‒PCR results verified that TGF-β1 stimulation significantly increased FSTL1 expression at both the mRNA and protein levels. Importantly, pretreatment with SB-431542 markedly attenuated this inductive effect (Fig. 7K & Supplementary Figure 4F, G).

**Fig. 8:**
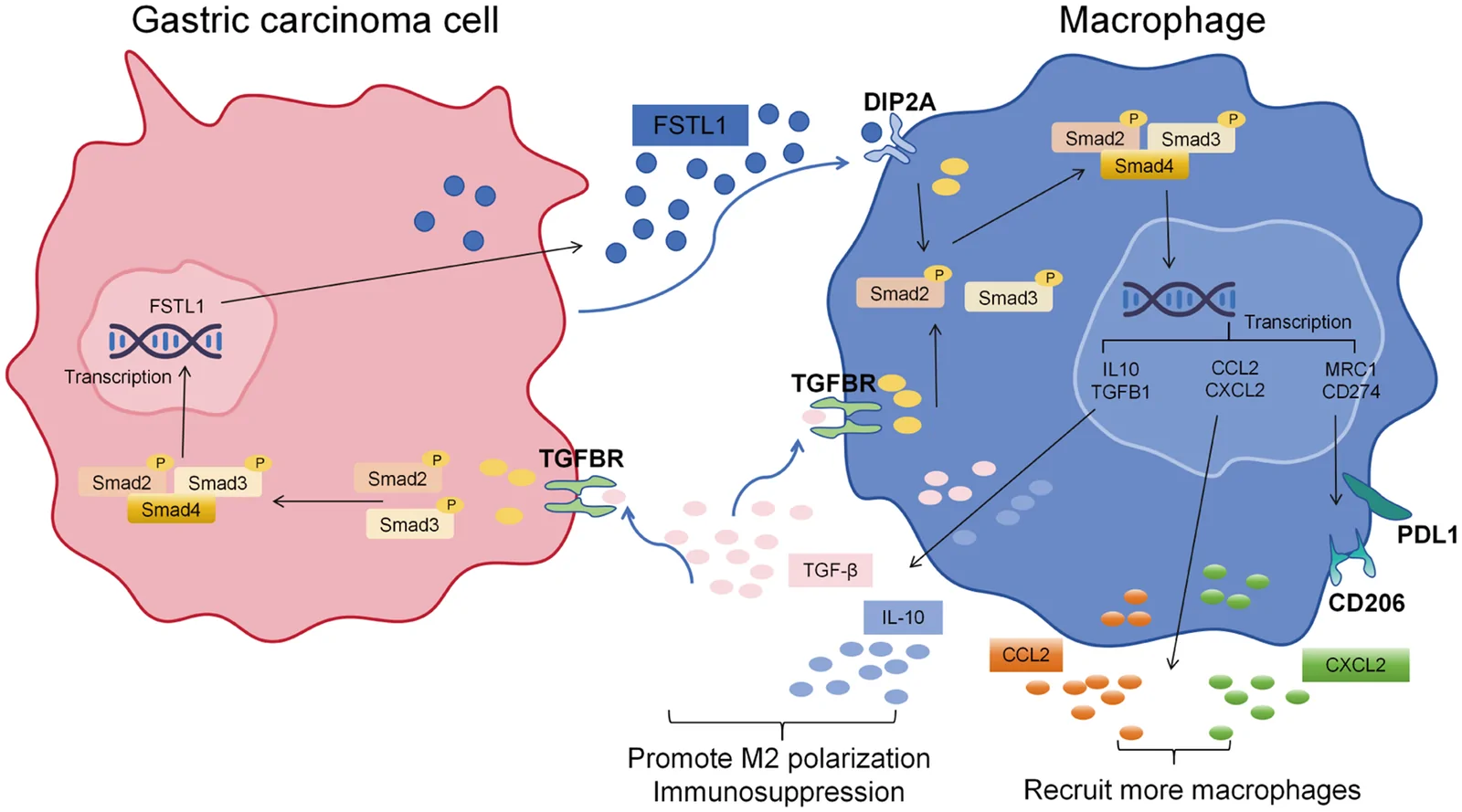
Schematic model of the molecular mechanism identified in this study. A schematic model illustrating that FSTL1 derived from gastric cancer cells binds to the macrophage surface receptor DIP2A, activates the TGF‑β/Smad2/3 pathway to promote M2 polarization, and establishes a GC cell–FSTL1–macrophage–TGF‑β transcellular positive feedback loop that reinforces the immunosuppressive TME and contributes to malignant progression

Collectively, our data support a model in which FSTL1 secreted by GC cells activates the TGF-β/Smad2/3 signaling pathway in macrophages to drive their polarization toward the M2 phenotype. Polarized M2 macrophages then secrete large amounts of TGF-β, which on the one hand acts as a classic M2 inducer to directly promote the M2 polarization of adjacent macrophages and, on the other hand, reciprocally induces GC cells to further upregulate FSTL1 expression. This leads to the sustained secretion of FSTL1 by GC cells, which in turn acts on macrophages, ultimately forming a transcellular positive feedback loop of “GC cells–FSTL1–macrophages–TGF-β” (Fig. 8). In support of this model, mIF staining of syngeneic tumor tissues revealed that *Fstl1* knockdown not only reduced M2 macrophage infiltration but also decreased intratumoral Tgfb1 levels, providing in vivo correlative evidence for the feedback loop.

## Discussion

As a secreted glycoprotein, FSTL1 exerts a tumor-type-dependent bidirectional regulatory effect on tumorigenesis and progression, and its oncogenic role in GC has been repeatedly validated [27, 28]. Previous work has demonstrated that FSTL1 reshapes immune cell infiltration to establish an immunosuppressive TME, yet few studies have systematically clarified its regulatory function in macrophage polarization [19, 21]. This current study extended the understanding of the mechanistic landscape of FSTL1 in GC immunity and characterized a novel immune evasion axis driven by FSTL1. Accumulating evidence has also indicated that context-dependent dual functions of FSTL1 across distinct malignancies, such as divergent pro- or antitumor activity, are likely determined by tumor cell origin, disease stage and local stromal composition, which we have elaborated in this study in the context of a GC-specific immune system.

While our study focused on tissue-derived FSTL1 in the TME, emerging clinical evidence supports the relevance of circulating FSTL1 levels. Kudo-Saito et al. recently demonstrated that high baseline serum FSTL1 levels in advanced GC patients were significantly associated with shorter progression-free survival (PFS) and overall survival (OS) following nivolumab monotherapy [20]. Notably, the combination of low baseline FSTL1 and low DIP2A^+^ cell levels in peripheral blood identified durable responders to anti-PD-1 therapy, suggesting that the FSTL1–DIP2A axis in the circulation may serve as a surrogate marker of the immunosuppressive TME. Although a direct comparison of serum FSTL1 concentrations between GC patients with GC and healthy individuals has not been reported to date, these clinical data suggest that elevated circulating FSTL1 levels may serve as a poor prognostic indicator in patients with GC. Systematic profiling of plasma levels of FSTL1 across disease stages and their correlation with tissue expression represents an important direction for future translational research.

Macrophage polarization within the TME is governed by a complex network of cytokines, metabolites and stromal-secreted factors, among which TGF-β, IL-6, lactic acid and CCL2 are well-established M2 inducers [29–35]. The present study focused primarily on canonical TGF-β/Smad2/3 signaling downstream of DIP2A, whereas noncanonical TGF-β branches and other transcription factors driving M2 polarization such as STAT3 and PPARγ, were are not fully examined in this study, which suggests important directions for follow-up research. FSTL1 likely interacts synergistically with these mediators to coordinate TAM reprogramming, and identifying this integrated regulatory network will reveal additional therapeutic targets to reverse M2 polarization in GC.

An acknowledged limitation of this work is the interspecies discrepancy between our in vitro and in vivo experimental systems. Human *FSTL1* and murine *Fstl1* exhibit 94.4% sequence conservation within mature functional domains critical for receptor binding, whereas sequence variation occurs mainly in the N-terminal signal peptide, which does not participate in DIP2A interactions [36]. Although published quantitative biochemical evidence directly comparing their binding affinity to DIP2A remains limited, the high structural conservation of the ligand-binding interface supports the biological relevance of our murine model. Moreover, the core intracellular signaling cascade downstream of DIP2A (TGF-β/Smad2/3) is highly conserved between human and mouse macrophages, providing a mechanistic rationale for extrapolating findings from THP-1 cells and human MDMs to the murine syngeneic model.

While our findings position FSTL1 as a promising candidate for immunomodulation, we acknowledge that its clinical translation requires further validation. We refrain from overstating the translational potential of FSTL1 as a standalone immunotherapeutic target, as immune checkpoint blockade combined with FSTL1 interference was not evaluated in the current study. Future in vivo combinatorial therapy experiments are warranted to determine whether targeting FSTL1 can synergize with anti-PD-1/PD-L1 regimens for gastric cancer treatment.

Despite these limitations, our study systematically characterized a transcellular tumor–macrophage positive feedback loop, clarified the mechanism underlying TAM-mediated immune suppression in GC, and provided new molecular insights for identifying therapeutic targets to reprogram the immunosuppressive TME of GC.

## Conclusion

The results of this study reveals the molecular mechanism by which FSTL1 drives M2 polarization of macrophages in GC through the DIP2A–Smad2/3 axis, and the transcellular positive feedback loop of “GC cells–FSTL1–macrophages–TGF-β”. These findings provide a theoretical basis and a immunomodulatory target candidate for targeting the immunosuppressive TME in GC.

## Electronic supplementary material

Below is the link to the electronic supplementary material.

Supplementary Material 1

Supplementary Material 2

Supplementary Material 3

Supplementary Material 4

Supplementary Material 5

Supplementary Material 6

## Data Availability

All the data generated or analyzed during this study are included in this published article and its supplementary information files.

## Abbreviations

FSTL1: Follistatin-like protein 1
GC: Gastric cancer
ICIs: Immune checkpoint inhibitors
TCGA: The Cancer Genome Atlas
STAD: Stomach adenocarcinoma
M2: M2-type macrophage
CD8^+^ T: CD8-positive T lymphocyte
DIP2A: Disco-interacting protein 2 homolog A
TGF-β: Transforming growth factor-β
Smad: Suppressor of mothers against decapentaplegic
TME: Tumor microenvironment
TAM: Tumor-associated macrophage
MDSCs: Myeloid-derived suppressor cells
PD-1: Programmed cell death protein 1
Tregs: Regulatory T cells
IL-10: Interleukin-10
NK cell: Natural killer cell
PD-L1: Programmed cell death ligand 1
CCL22: C-C motif chemokine ligand 22
RNA: Ribonucleic acid
LYZ: Lysozyme
CD68: Cluster of differentiation 68
MRC1: Mannose receptor C-type 1
ARG1: Arginase 1
C1QA: Complement component 1q A chain
CCL18: C-C motif chemokine ligand 18
C1QB: Complement component 1q B chain
UMAP: Uniform manifold approximation and projection
IHC: Immunohistochemical
DAB: 3,3’-diaminobenzidine
PBMCs: Peripheral blood mononuclear cells
FBS: Fetal bovine serum
MDMs: Monocyte-derived macrophages
PMA: Phorbol 12-myristate 13-acetate
siRNA: Small interfering RNA
shRNA: Short hairpin RNA
qRT–PCR: Quantitative real-time polymerase chain reaction
cDNA: Complementary DNA
GAPDH: Glyceraldehyde-3-phosphate dehydrogenase
WB: Western blotting
SDS-PAGE: Sodium dodecyl sulfate-polyacrylamide gel electrophoresis
HRP: Horseradish peroxidase
p-Smad2/3: Phosphorylated Smad2/3
ALK5: Activin receptor-like kinase 5
DMSO: Dimethyl sulfoxide
CCK-8: Cell Counting Kit-8
rhFSTL1: Recombinant human follistatin-like protein 1
Co-IP: Co-immunoprecipitation
DEG: Differentially expressed gene
GO: Gene Ontology
KEGG: Kyoto Encyclopedia of Genes and Genomes
GSEA: Gene set enrichment analysis
mIF: Multiplex immunofluorescence
TSA: Tyramide signal amplification
DAPI: 4’,6-diamidino-2-phenylindole
shNC: Short hairpin negative control
ELISA: Enzyme-linked immunosorbent assay
SD: Standard deviation
IQR: Interquartile range
PFS: Progression-free survival
OS: Overall survival
CCL2: C-C motif chemokine ligand 2
