# Supplementary material for: FSTL1 promotes M2 polarization of macrophages via the DIP2A–Smad2/3 Axis in gastric cancer
Source: J Transl Med. 2026 Aug 21;24:1303. doi: 10.1186/s12967-026-08836-1 (PMC13670623; doi:10.1186/s12967-026-08836-1)
Supplement: Supplementary file 1 [file 12967_2026_8836_MOESM5_ESM.docx]

**Table S1. qRT-PCR primer sequences used in this study**

| **Gene name** | **Primer forward sequence** | **Primer reverse sequence** |
| --- | --- | --- |
| *FSTL1* | CTCTAAAGGCAGCAACTACAGTG | CCCCTAAGCAACTTGTTGTTCTC |
| *GAPDH* | ACAACTTTGGTATCGTGGAAGG | GCCATCACGCCACAGTTTC |
| *Fstl1* | GGAATGTGCCGTCACAGAGAA | TGAGGTAGGTCTTGCCATTACT |
| *Gapdh* | AGGTCGGTGTGAACGGATTT | GGGGTCGTTGATGGCAACA |

**Table S2. siRNA sequences used in this study**

| **Name** | **Sense（5'-3'）** | **Antisense（5'-3'）** |
| --- | --- | --- |
| siNC | UUCUCCGAACGUGUCACGUTT | ACGUGACACGUUCGGAGAATT |
| si*FSTL1*-1 | GAAGAGCUAAGGAGCAAAUTT | AUUUGCUCCUUAGCUCUUCTT |
| si*FSTL1*-2 | CCUGGAGGAUGAAACGUAUTT | AUACGUUUCAUCCUCCAGGTT |
| si*FSTL1*-3 | CCAUGACCUGUGACGGAAATT | UUUCCGUCACAGGUCAUGGTT |
| si*DIP2A*-1 | GCACGUUAUAUACCGCUUATT | UAAGCGGUAUAUAACGUGCTT |
| si*DIP2A*-2 | GCGCUCGUGUUUCCGAAUATT | UAUUCGGAAACACGAGCGCTT |
| si*DIP2A*-3 | GCGUGUUAACAAGCGUCAUTT | AUGACGCUUGUUAACACGCTT |

**Table S3. shRNA target sequences used in this study**

| **Name** | **Target sequence** |
| --- | --- |
| shNC | TTCTCCGAACGTGTCACGT |
| Mus-*Fstl1*-sh1 | GCTGAAGATGCTGAGGACAAA |
| Mus-*Fstl1*-sh2 | GCAGATGAGTACCTGGAGTAT |
| Mus-*Fstl1*-sh3 | GGAGGAACCTAGAAGCAAATC |
